# Soft Tissue Sarcoma: New Paradigms in Care

**DOI:** 10.1155/2012/439208

**Published:** 2012-12-31

**Authors:** Peter Choong

**Affiliations:** ^1^Department of Surgery, University of Melbourne, St. Vincent's Hospital Melbourne, Victoria, Australia; ^2^Department of Orthopaedics, St. Vincent's Hospital Melbourne, Victoria, Australia; ^3^Bone & Soft Tissue Sarcoma Service, Peter MacCallum Cancer Centre, Victoria, Australia

Soft tissue sarcoma is a challenging tumour to treat not the least because its heterogeneity underlies a behaviour that is characterized by varying levels of local and systemic aggression. Moreover, its response to traditional modalities of treatment continues to confound surgeons as well as medical and radiation oncologists. This issue of Sarcoma sees the incorporation of work related to a number of key areas, which have drawn attention to how little is known about this tumour and the efforts currently being made to improve survival and progress disease control. 

J. M. Liberal et al. have explored the genetics of sarcomas to highlight the emerging field of targeted therapies. In their paper, they expand on the numbers of unique and typical molecular aberrations, which typify this class of tumour. They provide a considered perspective on how these aberrations provide novel targets for genetic and small molecule manipulation. A. Avdalyan et al. have examined the microvascularity of the tumour as a prognostic indicator. This underscores the importance of standard immunohistochemical approaches to delineating cellular characteristics that provide an insight into behaviour and thus prognosis. 

The very challenging area of functional assessment in adolescents and young adults is examined by M. Clayer et al. who have expanded on the well-described extremity scoring system from Toronto for analyzing postoperative functional assessment. M. H. Tang et al., who have identified quality of life assessments as a key area requiring further attention, complement this work. Indeed, the psychosocial aspect of response to sarcoma is an area poorly understood and yet critical because of the impact of disease that attacks the independence and mobility of young and middle-aged patients. 

Finally, the work by D. Grinsell et al. highlights the advances in soft tissue reconstructions and in particular, the role of innervated myocutaneous free flaps as functional reconstructions after wide resections. 

*Peter Choong* *Peter Choong*

